# Assessment and comparison of probability scores to predict giant cell arteritis

**DOI:** 10.1007/s10067-023-06721-6

**Published:** 2023-08-01

**Authors:** Chadi Sargi, Stephanie Ducharme-Benard, Valerie Benard, Rosalie-Selene Meunier, Carolyn Ross, Jean-Paul Makhzoum

**Affiliations:** 1 Department of Medicine, Montreal Sacre-Coeur Hospital, University of Montreal, Montreal, QC Canada; 2 Vasculitis Clinic, Department of Medicine, Montreal Sacre-Coeur Hospital, University of Montreal, Montreal, QC Canada; 3 Vasculitis Clinic, Canadian Network for Research on Vasculitides (CanVasc), Department of Medicine, Montreal Sacre-Coeur Hospital, University of Montreal, Montreal, QC Canada

**Keywords:** Diagnosis, Giant cell arteritis, Probability, Ultrasonography

## Abstract

**Introduction/objectives:**

To assess and compare the performance of the giant cell arteritis probability score (GCAPS), Ing score, Bhavsar-Khalidi score (BK score), color Doppler ultrasound (CDUS) halo count, and halo score, to predict a final diagnosis of giant cell arteritis (GCA).

**Method:**

A prospective cohort study was conducted from April to December 2021. Patients with suspected new-onset GCA referred to our quaternary CDUS clinic were included. Data required to calculate each clinical and CDUS probability score was systematically collected at the initial visit. Final diagnosis of GCA was confirmed clinically 6 months after the initial visit, by two blinded vasculitis specialists. Diagnostic accuracy and receiver operator characteristic (ROC) curves for each clinical and CDUS prediction scores were assessed.

**Results:**

Two hundred patients with suspected new-onset GCA were included: 58 with confirmed GCA and 142 without GCA. All patients with GCA satisfied the 2022 ACR/EULAR classification criteria. A total of 5/15 patients with GCA had a positive temporal artery biopsy. For clinical probability scores, the GCAPS showed the best sensitivity (Se, 0.983), whereas the BK score showed the best specificity (Sp, 0.711). As for CDUS, a halo count of 1 or more was found to have a Se of 0.966 and a Sp of 0.979. Combining concordant results of clinical and CDUS prediction scores showed excellent performance in predicting a final diagnosis of GCA.

**Conclusion:**

Using a combination of clinical score and CDUS halo count provided an accurate GCA prediction method which should be used in the setting of GCA Fast-Track clinics.

**Table:** 

**Key Points** • *In this prospective cohort of participants with suspected GCA, 3 clinical prediction tools and 2 ultrasound scores were compared head-to-head to predict a final diagnosis of GCA.* • *For clinical prediction tools, the giant cell arteritis probability score (GCAPS) had the highest sensitivity, whereas the Bhavsar-Khalidi score (BK score) had the highest specificity.* • *Ultrasound halo count was both sensitive and specific in predicting GCA.* • *Combination of a clinical prediction tool such as the GCAPS, with ultrasound halo count, provides an accurate method to predict GCA.*

**Supplementary Information:**

The online version contains supplementary material available at 10.1007/s10067-023-06721-6.

## Introduction

Giant cell arteritis (GCA) is the most common primary systemic vasculitis in adult patients [1]. GCA usually occurs over the age of 50 years, with a peak incidence at 80 years of age [2, 3]. The lifetime risk of developing GCA is 1% in women, 0.5% in men, and is more common in northern European countries [4]. GCA manifestations are often nonspecific and the various clinical phenotypes pose a diagnostic challenge. A new persistent headache is the most common symptom, occurring in 50–80% of cases [5]. Visual symptoms are reported in 15–26% of patients, with the dreaded complication of permanent visual loss mainly due to anterior ischemic optic neuropathy [7]. Rapid identification and treatment of GCA is therefore important to prevent permanent vision loss and contralateral eye involvement [8].

Temporal artery biopsy (TAB) is not available in all centers and its performance is highly variable. Prolonged use of glucocorticoids may influence TAB results. Furthermore, the presence of vasculitis may be missed due to the presence of segmental arteritis (skip lesions), irrespective of TAB length [9, 10]. Overall, false negative TAB is reported in 15–60% of patients with GCA [10–12].

Because TAB is not always accurate or available, clinical assessment of patients remains important [6]. Pre-test probability should be estimated to appropriately plan subsequent investigations, when required. If suspicion of GCA is high, empiric therapy with glucocorticoids should be initiated [13].

Many clinical probability tools have been developed in the last years to predict GCA (Table S1, supplementary data). The giant cell arteritis probability score (GCAPS) was published in 2019 and is simple to calculate without a computer [14]. Similarly, the Bhavsar-Khalidi score (BK score) is a point system that was developed based on clinical experience and literature review. The BK score stratifies the risk of GCA as low, intermediate, or high [15]. The Ing score consists of an online multivariate tool which takes into account clinical features and outputs a percentage risk as part of a stratum [16].

Color Doppler ultrasound (CDUS) of cranial and extra-cranial arteries is increasingly used in patients with suspected GCA. The presence of a hypoechoic circumferential intima-media thickening (halo sign) due to inflammation is consistent with the presence of vasculitis [17–19]. CDUS has excellent sensitivity (Se) and specificity (Sp) when performed by experienced clinicians with appropriate training [20]. CDUS is now the first-line diagnostic modality recommended in recent recommendations for the use of imaging in large-vessel vasculitis (LVV) [21]. Moreover, positive temporal or axillary artery CDUS is now part of the 2022 American College of Rheumatology/European League Against Rheumatism (ACR/EULAR) GCA classification criteria [22].

CDUS halo count (range from 0 to 8) refers to the total number of arterial branches (superficial, parietal, frontal temporal arteries, and axillary arteries) where a halo sign is found [23]. CDUS halo score (range from 0 to 48) is calculated by grading the maximum intima-media thickening (in millimeters) for each arterial branch (Table S2 and S3, supplementary data) [23, 24].

External validation of these clinical and CDUS prediction tools is required. Furthermore, no study has directly compared these tools head-to-head. The objective of this study is to assess and compare the performance of the GCAPS, Ing score, BK score, halo count, and halo score, to predict a final diagnosis GCA.

## Materials and methods

### Study design and patient population

We conducted a prospective cohort study at our quaternary vasculitis clinic from April to December 2021. To be included, participants had to be (i) adult patients over 18 years of age, (ii) referred to our GCA Fast-Track clinic with a suspicion of new-onset GCA, and (iii) provide informed written consent to the study. Patients were excluded if they were (i) referred for a GCA relapse, (ii) currently taking chronic glucocorticoids (at any dose, currently and for more than 30 consecutive days) or immunosuppressive therapy (current use and for more than 30 consecutive days), and (iii) if they had a TAB performed prior to CDUS.

### Clinical data elements

At the initial visit, participants underwent a complete clinical assessment, physical examination, and had a CDUS of temporal and axillary arteries. A standardized case report form (CRF) was used to document every clinical and CDUS element required to calculate probability scores. Data collected also included patient characteristics, past medical history, clinical presentation, detailed physical examination, previous bloodwork (prior or within 72 h of glucocorticoid initiation), cumulative glucocorticoid dose, and whether an alternative diagnosis to GCA was considered.

### Color Doppler ultrasound of cranial and axillary arteries

During that same visit, CDUS was performed by one of three experimented GCA specialists (VB, SDB, JPM) using a Canon Xario^TM^ 200 Platinum series with an 18L7 probe for cranial arteries, and a 14L5 probe for axillary arteries. All branches of temporal arteries (common superficial, frontal, parietal) were scanned in longitudinal and transverse planes, using two-dimensional grayscale ultrasound with and without color Doppler. Axillary arteries were scanned using the same technique. Performance and external validation of the CDUS equipment, technique, and ultrasound cut-off values for positivity had already been performed with results published [11]. Halo sign was defined as a hypoechoic circumferential intima-media complex, with a thickening of at least 0.4mm, 0.3mm, 0.3mm, and 1.0mm for the common superficial temporal artery, frontal temporal artery, parietal temporal artery, and axillary artery, respectively. The presence of a halo sign was confirmed by the inability to compress the artery (compression sign). Intima-media complex was quantitatively measured in each arterial branch at the site of maximal thickness.

### Confirmation of GCA diagnosis and blinding

Final diagnosis of GCA was confirmed clinically by two independent vasculitis specialists, 6 months after the initial visit. The physicians confirming GCA were blinded to the CRF containing CDUS and clinical prediction tools. GCA was confirmed in the presence of unequivocal symptoms, bloodwork, investigations, clinical evolution, and response to therapy. In patients without GCA, alternative diagnoses were investigated and documented.

### Clinical and CDUS probability scores

Every item required to calculate clinical and CDUS probability scores was collected at the initial visit. GCAPS, Ing score, BK score, halo count, and halo scores were officially calculated six months following the initial visit, using the CRF containing data systematically collected for that purpose. Scores were calculated by two independent investigators, blinded to the final diagnosis.

### Statistical analyses

Descriptive statistics were calculated for patient characteristics. When appropriate, statistical tests were performed using chi-squared test for categorical variables and independent samples *t*-test for continuous variables. A significance level of 0.05 was used. Receiver operator characteristic (ROC) curve was plotted for each clinical and ultrasound probability score and the best cut-off value was determined. Area under the curve (AUC) was calculated for each ROC analysis. Se, Sp, positive predictive value (PPV), and negative predictive value (NPV) were assessed for GCAPS, Ing score, BK score, halo count, and halo score individually. Furthermore, the combination of clinical scores with CDUS scores was evaluated using logistic regression models and combined ROC analyses. Paired-sample area difference under the ROC curves was calculated for each combination of clinical-CDUS score. Analyses were performed using Stata (StataCorp LLC) SE V17.0.

### Ethics approval

The study (protocol number 2020-1890) was approved by the scientific committee and research ethics board of Montreal Sacre-Coeur Hospital. Patients provided written informed consent. The study was performed in accordance with the principles of the Declaration of Helsinki and Good Clinical Practice guidelines.

## Results

**Fig. 1:**
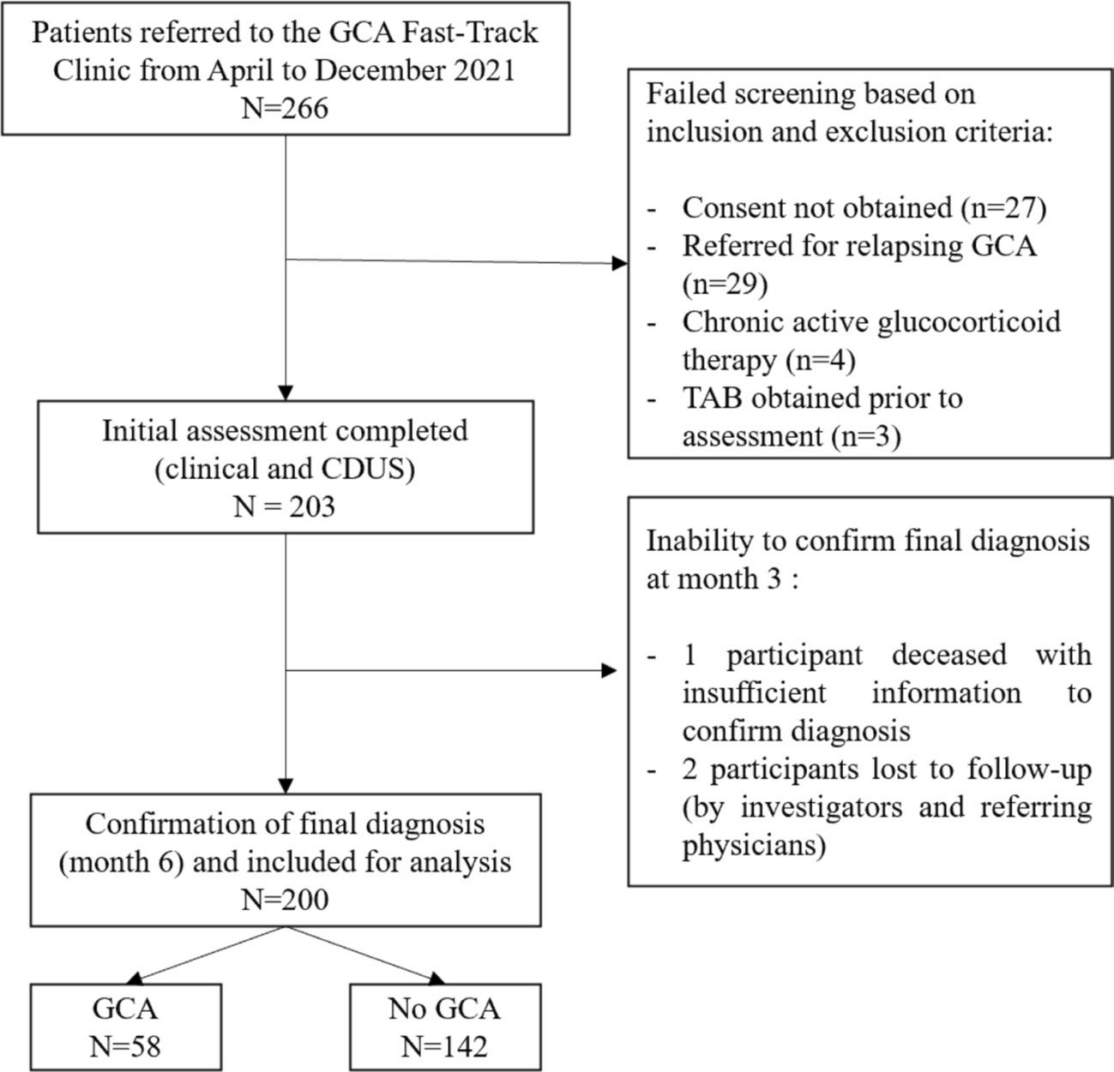
Flowchart of participants referred for suspected GCA. GCA, giant cell arteritis; TAB, temporal artery biopsy; CDUS, color Doppler ultrasound

Out of the 266 patients referred to our Fast-Track clinic, 200 were included for analysis: 58 with confirmed GCA and 142 without GCA (Fig. 1). All 58 patients with GCA satisfied the official 2022 ACR/EULAR classification criteria for GCA. Out of 15 patients who had a TAB, 5 of them were consistent with GCA. Baseline characteristics, clinical features, laboratory findings, and investigation modalities are detailed in Table 1. An alternative diagnosis was established in most patients without GCA (Table S4, supplementary data).

**Table 1:** Baseline characteristics and clinical presentation of 200 patients referred for suspected GCA

	GCA (*n*= 58)	No GCA (*n*= 142)
Demographic characteristics		
Age, mean (SD)	77.4 (8.6)	73.1 (9.7)
Female subjects, *n* (%)	33 (56.9)	99 (69.7)
White, *n* (%)	56 (96.6)	127 (89.4)
Previous medical history, *n* (%)		
CAD	9 (15.5)	21 (14.8)
PAD	10 (17.2)	16 (11.3)
HTN	33 (56.9)	93 (65.5)
Dyslipidemia	25 (43.1)	65 (45.8)
Diabetes	10 (17.2)	37 (26.1)
CKD	9 (15.8)	25 (17.6)
GCA symptoms and signs, *n *(%)		
New headache	45 (77.6)	108 (76.1)
Scalp tenderness	27 (46.5)	35 (24.6)
PMR	13 (22.4)	10 (7.0)
Weight loss	21 (36.2)	23 (16.2)
Fatigue	31 (53.4)	42 (29.6)
Hyperthermia	9 (15.5)	14 (9.9)
Jaw claudication	24 (41.4)	2 (1.4)
Vision loss	13 (22.4)	17 (12.0)
Amaurosis fugax	6 (10.3)	7 (4.9)
Diplopia	11 (19.0)	14 (9.9)
Abnormal TA exam^a^	44 (75.9)	15 (10.6)
Asymmetrical limb BP^b^	6 (10.3)	20 (14.1)
Abnormal ophthalmic exam^c^	17/36 (47.2)	20/85 (23.5)
Cranial nerve palsy	5 (8.6)	5 (3.5)
Laboratory findings, mean (SD)		
WBC (x 10^9/L)	8.7 (3.5)	8.3 (2.9)
Hb (g/L)	121.1 (16.2)	127.8 (17.4)
PLT (x 10^9/L)	321.3 (139.0)	283.1 (109.7)
ESR (mm/hr)	51.65 (23.6)	34.2 (23.2)
CRP (mg/L)	64.9 (71.0)	32.3 (51.4)
GCA investigation and therapy		
Positive TAB, *n* (%)	5/15 (33.3)	0/8 (0)
Positive CDUS, *n* (%)	56 (96.6)	3 (2.1)
Empirical GC initiated, *n* (%)	47 (81.0)	77 (54.2)
Days on empirical GC, mean (SD)^d^	5.0 (6.1)	3.6 (5.9)
Cumulative GC in mg, median (IQR)^d^	240 (60–450)	120 (0–300)

*GCA* giant cell arteritis, *SD* standard deviation, *CAD* coronary artery disease, *PAD* peripheral artery disease, *HTN* hypertension, *CKD* chronic kidney disease, *PMR* polymyalgia rheumatica, *TA* temporal arteries, *BP* blood pressure, *WBC* white blood count, *Hb* hemoglobin, *PLT* platelet, *ESR* erythrocyte sedimentation rate, *CRP* C-reactive protein, *TAB* temporal artery biopsy, *CDUS* color Doppler ultrasound, *GC* glucocorticoids, *IQR* interquartile range

^a^Abnormal temporal arteries defined as either pulselessness, reduced pulse, thickening or tenderness on palpation

^b^Defined as a difference of more than 10 mmHg in systolic blood pressure

^c^Performed by an ophthalmologist. Includes anterior ischemic optic neuropathy, central retinal occlusion, reverse afferent pupillary defect, visual field abnormalities

^d^Number of days and doses (mg) in prednisone equivalent at the first clinical Fast-Track clinic assessment (where clinical probability of GCA assessment and CDUS was performed)

### Performance of clinical probability scores

Established cut-off values considered positive were > 9.5 points for GCAPS, 14% cut point for Ing score (level 3 or more), and ≥ 5 points for BK score (moderate probability or more).

**Fig. 2:**
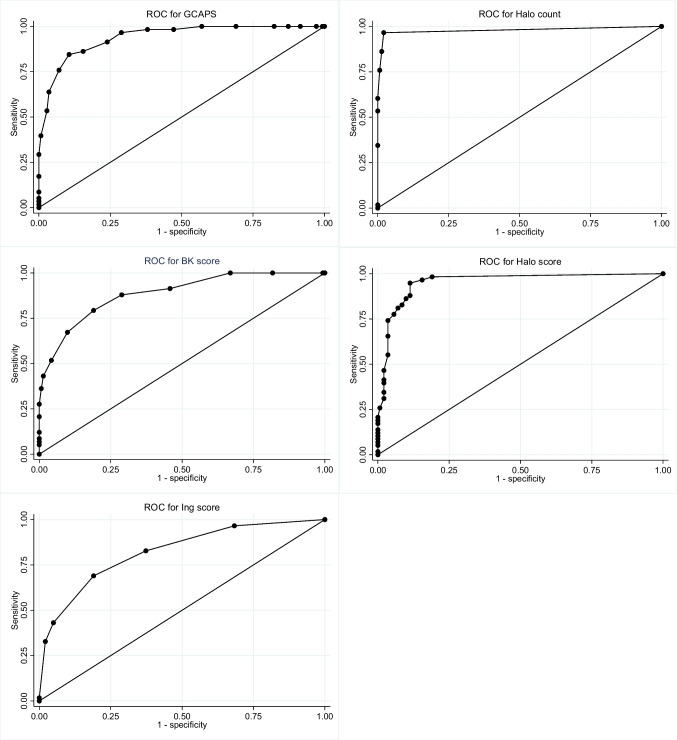
Receiver operating characteristic for clinical and ultrasound probability tools. ROC, receiver operating characteristic; GCAPS, giant cell arteritis probability score; BK score, Bhavsar-Khalidi score

ROC AUC was excellent for the GCAPS and good for both Ing score and BK score (Fig. 2, Table 2). GCAPS had the highest Se: 0.983 (95% confidence interval [95%CI]: 0.908–0.999), while BK score had the highest Sp of 0.711 (95%CI: 0.629–0.784). NPV was highest for GCAPS followed by BK score, respectively, 0.989 (95% CI: 0.926–0.998) and 0.935 (95% CI: 0.877–0.969) (Table 2). The accuracy of each clinical probability score (probability of correct classification) was 0.725, 0.685 and 0.760 for the GCAPS, Ing score, and BK score, respectively.

**Table 2:** Performance of clinical and ultrasound probability tools to predict a final diagnosis of GCA in 200 patients assessed for suspected GCA

	Probability tools (95% CI)				
	GCAPS^a^	Ing^b^	BK score^c^	Halo count^d^	Halo score^e^
Se	0.983 (0.908–0.999)	0.828 (0.706–0.914)	0.879 (0.767–0.950)	0.966 (0.881–0.996)	0.966 (0.881–0.996)
Sp	0.619 (0.535–0.699)	0.626 (0.542–0.706)	0.711 (0.629–0.784)	0.979 (0.939–0.996)	0.845 (0.775–0.900)
PPV	0.514 (0.460–0.566)	0.475 (0.415–0.536)	0.554 (0.486–0.621)	0.949 (0.859–0.983)	0.712 (0.634–0.789)
NPV	0.989 (0.926–0.998)	0.899 (0.833–0.941)	0.935 (0.877–0.969)	0.986 (0.947–0.996)	0.984 (0.939–0.996)
LR+	2.58 (2.09–3.20)	2.22 (1.74–2.83)	3.05 (2.31–4.01)	45.70 (14.90–140.16)	6.23 (4.23–9.18)
LR-	0.03 (0.00–0.20)	0.28 (0.15–0.49)	0.17 (0.08–0.34)	0.04 (0.01–0.14)	0.04 (0.01–0.16)
ROC AUC	0.9391 (0.8975–0.9686)	0.8214 (0.7570–0.8849)	0.8861 (0.8368–0.9354)	0.9789 (0.9544–1.0000)	0.9533 (0.9236–0.9830)
Accuracy	0.725 (0.658–0.786)	0.685 (0.616–0.749)	0.760 (0.695–0.817)	0.975 (0.943–0.992)	0.880 (0.827–0.922)

*CI* confidence interval, *GCAPS* giant cell arteritis probability score, *BK score* Bhavsar-Khalidi score, *Se* sensitivity, *Sp* specificity, *PPV* positive predictive value, *NPV* negative predictive value, *LR+* positive likelihood ratio, *LR-* negative likelihood ratio, *ROC AUC* receiver operating characteristics area under curve

^a^GCAPS cut-off > 9.5

^b^Ing score cut-off ≥ level 3

^c^BK score cut-off ≥ 5

^d^Halo count cut-off ≥ 1

^e^Halo score cut-off ≥ 2

### Performance of CDUS probability scores

Established cut-off values considered positive were ≥ 1 for halo count, and ≥ 2 for halo score. Seven participants had a halo count of 1 (only one abnormal arterial segment with halo sign). GCA was the final diagnosis in six of those participants.

Both CDUS scores performed well, with an excellent ROC AUC of 0.979 (95% CI: 0.954–1.000) for halo count and 0.953 (95% CI: 0.923–0.983) for halo score (Fig. 2). Se was 0.966 (95% CI: 0.881–0.996) for both halo count and halo score. Sp was 0.979 (95% CI: 0.939–0.996) and 0.845 (95%CI: 0.775–0.900) for halo count and halo score, respectively (Table 2). Positive likelihood ratio (LR+) was 45.7 (95% CI: 14.9–140.2) and 6.2 (95% CI: 4.2–9.2), for halo count and halo score, respectively. The accuracy (probability of correct classification) of halo count was 0.975 and 0.880 for halo score.

### Combination of clinical and CDUS score to predict GCA

Classification of patients using a combination of clinical scores and CDUS halo count is presented in Table 3. Combination of GCAPS and CDUS halo count showed the best overall classification accuracy. In patients with GCA, none had a normal GCAPS/normal halo count combination. GCAPS allowed the identification of GCA in two patients with a negative halo count. Logistic regression models and paired-sample area difference under the ROC curves showed that the performance of this combination is mainly driven by the halo count (Table S5 and S6, supplementary data).

**Table 3:** Classification of participants using a combination of clinical and ultrasound probability tools to predict a final diagnosis of GCA

Number of patients, *n *(%)	GCA *n=*58	No GCA *n=*142
GCAPS +^a^ / Halo count +^d^	55 (94,8)	2 (1.4)
GCAPS + / Halo count -	2 (3.5)	52 (36.6)
GCAPS - / Halo count +	1 (1.7)	1 (0.7)
GCAPS - / Halo count -	0	87 (61.3)
Ing score +^b^/ Halo count +	47 (80.0)	2 (1.4)
Ing score + / Halo count -	1 (1.7)	51 (35.9)
Ing score - / Halo count +	9 (15.5)	1 (0.7)
Ing score - / Halo count -	1 (1.7)	88 (62.0)
BK score +^c^ / Halo count +	50 (86.2)	2 (1.4)
BK score + / Halo count -	1 (1.7)	39 (27.5)
BK score - / Halo count +	6 (10.4)	1 (0.7)
BK score - / Halo count -	1 (1.7)	100 (70.4)

*GCA* giant cell arteritis, *GCAPS* giant cell arteritis probability score, *BK score* Bhavsar-Khalidi score

^a^GCAPS cut-off > 9.5

^b^Ing score cut-off ≥ level 3

^c^BK score cut-off ≥ 5

^d^Halo count cut-off ≥ 1

## Discussion

This prospective cohort study directly compared clinical and CDUS prediction scores in GCA. Moreover, every patient with a final diagnosis of GCA satisfied the 2022 ACR/EULAR classification criteria.

The three clinical prediction scores are easy to calculate. The GCAPS and BK score do not require a calculator or spreadsheet. The GCAPS was found to have the highest Se and lowest negative likelihood ratio (LR-). This is comparable to data previously published [11, 14]. With a GCAPS < 9.5 points, clinicians may therefore feel comfortable in excluding a diagnosis of GCA. The BK score showed the highest specificity and PPV at the established cut-off value. However, similarly to the GCAPS and Ing score, a positive score only mildly increased (LR+ 3.1) the probability of GCA. This highlights the wide range of nonspecific symptoms in GCA and its large differential diagnosis.

The performance of CDUS scores was excellent; however, halo count had the best PPV and LR+. Halo count requires subjective interpretation of vessel wall echogenicity. It is calculated by counting arterial branches where a hypoechoic circumferential intima-media thickening (halo sign) is present. Halo sign has previously proven to be specific in confirming the presence of vasculitis. It is a visual representation of active inflammation within the vessel wall [23]. Threshold values of intima-media complex defining a halo sign is debated amongst GCA experts and may vary according to the equipment used. The values used in this study are widely recognized and have been externally validated in our center in a previous study [11]. In contrast, halo score is purely quantitative and involves measuring and grading the maximal intima-media thickness for each arterial branch. However, thickening of the vessel wall may occur in the presence of atherosclerosis (isoechoic or hyperechoic) and increases the halo score. Halo score may therefore be less specific, and increased in the absence of active, hypoechoic, vessel wall inflammation.

High doses of glucocorticoids may affect CDUS prediction scores by reducing vessel wall inflammation [25, 26]. In our cohort, a higher proportion of patients with GCA had empirical glucocorticoids initiated before study inclusion (Table 1). Moreover, the cumulative glucocorticoid dose was double in participants with GCA compared to those without GCA. The performance of halo count and halo score may therefore have been underestimated.

Combining the GCAPS with halo count allowed correct identification of all patients with GCA. Thus, GCA may be excluded with confidence in the setting of a low clinical suspicion of GCA on the GCAPS combined with a normal halo count on CDUS.

Based on the CDUS halo count alone, only 5 patients were misclassified: 2 with a false negative (FN) and 3 with a false positive (FP) result. In one patient with a FN halo count, all three clinical scores were positive. In the other patient with a FN halo count, only the GCAPS was positive and allowed prediction of GCA. Both patients with FN halo count also had a negative TAB but had evidence of extra-cranial GCA on large vessel imaging. In the three patients with FP halo count, one patient had negative clinical scores, while the other two patients had positive clinical scores. The two patients with FP clinical scores and halo count both had a negative TAB and an alternate diagnosis at follow-up (head and neck pathology, and viral infection).

TAB was not required in our protocol as it is no longer considered a reference test in most GCA studies. Prospective clinical follow-up of participants to confirm GCA was performed, which is now routinely used in GCA diagnostic accuracy studies. Using composite reference tests (CDUS, cranial MRA, cranial PET-CT, large vessel imaging) combined with prospective clinical follow-up could improve the certainty of a final diagnosis of GCA. This is often difficult to perform in studies due to limited availability of tests and required expertise, cost, and the risks associated with TAB and/or cumulative radiation exposure.

Our study had several strengths. We included a large number of participants and assessed five GCA prediction tools simultaneously. Data collection on prediction scoring items was performed prospectively to reduce recall bias. Final diagnosis of GCA was performed by two investigators, independently, without knowledge of prediction scores to avoid an overestimation of effect.

Our study also had limitations. Not every patient had a formal ophthalmologic examination, which is a scoring item in the GCAPS. Patients without any visual symptoms were attributed a score of 0 for that GCAPS item in the absence of a formal retinal examination. Although unlikely, subclinical retinal changes may have been missed and would have resulted in a higher GCAPS. However, patients without visual symptoms are usually not referred to ophthalmologists and this represents “real life” practice for most clinicians.

In conclusion, many clinical and CDUS prediction scores are available to predict GCA. GCAPS was the clinical prediction score with the highest sensitivity, whereas the BK score had the highest specificity. The performance of CDUS prediction scores, mostly the halo count, is excellent. Combining GCAPS and halo count allowed correct identification of all patients with GCA and should be considered in the setting of GCA Fast-Track clinics.

### Supplementary information

ESM 1 (DOCX 36.5 KB)

## Data Availability

Dr. Makhzoum confirms the accuracy, completeness, and availability of the data. The datasets generated during and/or analyzed during the current study are available from the corresponding author on reasonable request.
