# Supplementary material for: Assessment and comparison of probability scores to predict giant cell arteritis
Source: Clin Rheumatol. 2023 Aug 1;43(1):357–65. doi: 10.1007/s10067-023-06721-6 (PMC10774184; doi:10.1007/s10067-023-06721-6)
Supplement: Supplementary file 1 — ESM 1 (DOCX 36.5 KB) [file 10067_2023_6721_MOESM1_ESM.docx]

Assessment and Comparison of Probability Scores to Predict Giant Cell Arteritis

Chadi Sargi, Stephanie Ducharme-Benard, Valerie Benard, Rosalie-Selene Meunier,
Carolyn Ross, Jean-Paul Makhzoum

**Supplementary Data**

Table of contents

Table S1 – Items scored in the clinical probability scores p2

Table S2 – Ultrasound halo count calculation p3

Table S3 – Ultrasound halo score calculation p4

Table S4 – Alternate diagnosis in participants without GCA p5

Table S5 – Logistic regression models to predict GCA p6

Table S6 – Paired-sample area difference under the ROC curves p7

**Corresponding author:**

Jean-Paul Makhzoum, MD, FRCPC, FACP

ORCID**: 0000-0003-4523-8525**

Associate Professor

Vasculitis Clinic, Department of Medicine

Canadian Network for Research on Vasculitides

Montreal Sacre-Cœur Hospital

University of Montreal

Tel:514-338-2725

Fax: 514-338-2789

[jean-paul.makhzoum@umontreal.ca](mailto:jean-paul.makhzoum@umontreal.ca)

**Supplementary Table S1** – Items scored in the clinical probability tools.

| **Clinical probability tools** | **Item scored** |
| --- | --- |
| GCAPS (22) | Age  Gender  Duration since onset of symptoms  CRP (mg/L)  Headache  Polymyalgia rheumatica  Constitutional symptoms  Ischemic symptoms  Visuals signs (AION, CRAO Field loss, RAPD)  Abnormal temporal arteries on examination  Extra-cranial artery abnormality  Cranial nerve palsy  Presence of alternative diagnosis: infection, cancer, other rheumatic disease, head and neck pathology, other. |
| BK score (23) | Typical headache  Atypical headache  Scalp tenderness  Jaw claudication  Sudden visual loss  Other visual symptoms  Polymyalgia symptoms  Constitutional symptoms  Temporal artery tenderness  Temporal artery decreased pulse  ESR or CRP |
| Ing score (24) | Age  Gender  New headache  Temporal artery tenderness or reduction in pulse  Jaw-tongue claudication  Vision loss (AION, PION, CRAO)  Diplopia  ESR  CRP  Upper limit of normal CRP in your lab  Platelet level |

GCAPS (Giant cell arteritis probability score), BK score (Bhavsar-Khalidi score), CRP (C-reactive protein), AION (anterior ischemic optic neuropathy), CRAO (central retinal artery occlusion), RAPD (relative afferent pupillary defect), PION (posterior ischemic optic neuropathy), ESR (erythrocyte sedimentation rate)

**Supplementary Table S2** – Ultrasound halo count documentation in participants
with suspected Giant cell arteritis

| Arterial Segment | Check box if there is at least one halo sign in that arterial segment |
| --- | --- |
| Right superficial temporal artery |  |
| Left superficial temporal artery |  |
| Right parietal temporal artery |  |
| Left parietal temporal artery |  |
| Right frontal temporal artery |  |
| Left frontal temporal artery |  |
| Right axillary artery |  |
| Left axillary artery |  |
| Total number of checked boxes | Halo count: ______ / 8 |

**Supplementary Table S3** – Ultrasound halo score calculation in participants with suspected GCA

| Arterial Segment | Points according to intima-media complex measurement^s^ | Result  (points) |
| --- | --- | --- |
| Right superficial temporal artery | ≤ 0.3 mm 🡪 0 point  0.4 mm 🡪 1 point  0.5 mm 🡪 2 points  0.6 to 0.7 mm 🡪 3 points  ≥ 8 mm 🡪 4 points | __ / 4 |
| Left superficial temporal artery | ≤ 0.3 mm 🡪 0 point  0.4 mm 🡪 1 point  0.5 mm 🡪 2 points  0.6 to 0.7 mm 🡪 3 points  ≥ 0.8 mm 🡪 4 points | __ / 4 |
| Right parietal temporal artery | ≤ 0.2 mm 🡪 0 point  0.3 mm 🡪 1 point  0.4 mm 🡪 2 points  0.5 mm 🡪 3 points  ≥ 0.6 mm 🡪 4 points | __ / 4 |
| Left parietal temporal artery | ≤ 0.2 mm 🡪 0 point  0.3 mm 🡪 1 point  0.4 mm 🡪 2 points  0.5 mm 🡪 3 points  ≥ 0.6 mm 🡪 4 points | __ / 4 |
| Right frontal temporal artery | ≤ 0.1 mm 🡪 0 point  0.2 mm 🡪 1 point  0.3 mm 🡪 2 points  0.4 mm 🡪 3 points  ≥ 0.5 mm 🡪 4 points | __ / 4 |
| Left frontal temporal artery | ≤ 0.1 mm 🡪 0 point  0.2 mm 🡪 1 point  0.3 mm 🡪 2 points  0.4 mm 🡪 3 points  ≥ 0.5 mm 🡪 4 points | __ / 4 |
| Right axillary artery | ≤ 0.5 mm 🡪 0 point  0.6 mm 🡪 3 points  0.7 to 0.8 mm 🡪 6 points  0.9 to 1.5 mm 🡪 9 points  ≥ 1.6 mm 🡪 12 points | __ / 12 |
| Left axillary artery | ≤ 0.5 mm 🡪 0 point  0.6 mm 🡪 3 points  0.7 to 0.8 mm 🡪 6 points  0.9 to 1.5 mm 🡪 9 points  ≥ 1.6 mm 🡪 12 points | __ / 12 |
| TOTAL |  | ____ / 48 |

^a^ Points are attributed according to the site of highest intima-media complex measured in each
arterial segment.

**Supplementary Table S4** – Final alternate diagnosis in
142 participants without GCA

| **Alternate diagnosis** | **N** | **(%)** |
| --- | --- | --- |
| **Infection** |  |  |
| URTI | 5 | (3.5) |
| COVID-19 | 3 | (2.1) |
| Sinusitis | 2 | (1.41) |
| VZV | 1 | (0.7) |
| Pneumonia | 1 | (0.7) |
| Meningitis | 1 | (0.7) |
| Sepsis | 2 | (1.4) |
| **Musculoskeletal-mechanical** |  |  |
| TMJ | 5 | (3.5) |
| Neck | 5 | (3.5) |
| Shoulders | 2 | (1.4) |
| Hips | 2 | (1.4) |
| **Neurological** |  |  |
| Tension type headache | 64 | (45.1) |
| Migraines | 14 | (9.9) |
| Ischemic stroke | 2 | (1.4) |
| **Other** |  |  |
| Metastatic neoplasm | 3 | (2.1) |
| Uveitis | 1 | (0.7) |
| **Unknown/resolved symptoms^a^** | 29 | (20.4) |

URTI (upper respiratory tract infection), COVID-19 (coronavirus disease 2019),
VZV (varicella-zoster virus), TMJ (temporomandibular joint)

^a^ On initial assessment (month 0) no clear diagnosis was established, however all
symptoms resolved by month 6, without requiring any giant cell arteritis therapy.

**Supplementary Table S5** – Logistic regression models to predict GCA

| **Independent variable** | **b** | **SE** | **Z ratio** | **p** | **95% CI** |
| --- | --- | --- | --- | --- | --- |
| *Model – GCAPS category (>9.5) and halo count (*≥1) | | | | | |
| GCAPS > 9.5 | 8.616 | 10.846 | 0.79 | 0.427 | -12.642 - 29.874 |
| Halo count | 2.777 | 0.676 | 4.11 | < 0.001 | 1.453 - 4.101 |
| Constant | -11.255 | 10.904 | -1.03 | 0.302 | -32.625 - 10.116 |
| *Model – Ing category (level 3 or more) and halo count (*≥1) | | | | | |
| Ing level 3 or more | 1.324 | 0.885 | 1.50 | 0.135 | -0.411 - 3.058 |
| Halo count | 2.695 | 0.546 | 4.94 | <0.001 | 1.625 - 3.764 |
| Constant | -4.216 | 0.795 | -5.30 | <0.001 | -5.773 - (-2.658) |
| *Model – BK score category (intermediate or more) and halo count (*≥1) | | | | | |
| BK score (≥ 5) | 1.062 | 0.818 | 1.30 | 0.194 | -0.541 - 2.664 |
| Halo count | 2.686 | 0.555 | 4.84 | <0.001 | 1.599 - 3.773 |
| Constant | -3.928 | .659 | -5.96 | <0.001 | -7.290 - 2.637 |

GCA (Giant cell arteritis), GCAPS (Giant cell arteritis probability score), BK score (Bhavsar-Khalidi score), CI (confidence interval)

| **Test Result Pair(s)** | **AUC Difference** | **SE Difference** | **Z** | **p** | **95% CI** |
| --- | --- | --- | --- | --- | --- |
| **Halo count with:** |  |  |  |  |  |
| GCAPS | -0.011 | 0.137 | -1.314 | 0.189 | -0.028 - 0.006 |
| Ing score | -0.001 | 0.165 | -0.095 | 0.924 | -0.017 - 0.015 |
| BK score | -0.008 | 0.150 | -0.909 | 0.364 | -0.025 - 0.009 |

**Supplementary Table S6** – Paired-sample area difference under the ROC curves.

ROC (receiver operating characteristic), AUC (area under curve), SE (standard error), CI (confidence interval), GCAPS (Giant cell arteritis probability score), BK score (Bhavsar-Khalidi score)
